# SorCS3 suppresses adrenocortical carcinoma progression by enhancing IGF2R-mediated endocytic trafficking and signaling attenuation

**DOI:** 10.1186/s12967-025-07146-2

**Published:** 2025-10-21

**Authors:** Yanqiu Zhang, Shihui Cao, Jingqi Nie, Shengmiao Yu, Jinlai Gao, Shi Zhang, Feifei Zheng, Shipeng Bo, Nan Wang, Haiyan Xu, Yuchen Zhao, Chao Shen, Yang Li, Dongliang Li

**Affiliations:** 1 From the Department of Basic Medical College, Harbin Medical University (Daqing), Daqing, China; 2 Tianjin Medical University Cancer Institute and Hospital, Tianjin, China; 3 The College of Medical Laboratory Science and Technology, Harbin Medical University (Daqing), Daqing, China; 4 College of Medical, Jiaxing University, Jiaxing, China

**Keywords:** SorCS3, Adrenocortical carcinoma, Biomarker, Prognosis

## Abstract

**Background:**

Adrenocortical carcinoma (ACC) is a rare and aggressive malignancy with limited treatment options and poor prognosis. Identifying novel molecular regulators is essential for improving therapeutic strategies. In this study, we identified Sortilin-related VPS10 domain-containing receptor 3 (SorCS3) as a novel tumor suppressor candidate in ACC.

**Methods:**

Through expression detection and functional validation, we investigated the role of SorCS3, a member of the VPS10p-domain receptor family, in ACC. Expression data from TCGA and clinical tissue samples were analyzed. Endocytic activity, protein interactions, and downstream signaling were examined using immunofluorescence, co-immunoprecipitation (Co-IP), and western blotting in ACC cell lines.

**Results:**

SorCS3, previously regarded as CNS-specific, was detected in ACC tissues and cell lines. Low SorCS3 expression was associated with reduced overall survival (OS) and disease-specific survival (DSS). Functional assays showed that SorCS3 enhanced endocytosis and colocalized with early endosomes. Co-IP revealed a physical or indirect interaction between SorCS3 and Insulin-like growth factor 2 receptor (IGF2R), a multifunctional receptor involved in IGF-II degradation and signal attenuation. SorCS3 overexpression (OE-SorCS3) increased IGF2R protein levels and suppressed PI3K/Akt and MAPK/Erk signaling. Blocking endocytosis partially reversed these effects, supporting a receptor trafficking-dependent mechanism.

**Conclusions:**

In this study, we identified SorCS3 as a novel tumor suppressor candidate in ACC. Although previously thought to be CNS specific, SorCS3 was found to be expressed in ACC tissues and cell lines. Low SorCS3 levels correlated with poor patient survival in the TCGA cohort. Functionally, SorCS3 enhanced endocytosis in ACC cells and physically or indirectly interacted with IGF2R. OE-SorCS3 increased IGF2R protein levels and reduced phosphorylation of Akt/Erk, suggesting tumor-suppressive effects through IGF2R stabilization and signaling inhibition. Overall, our findings highlight the SorCS3-IGF2R axis as a previously unrecognized regulatory mechanism in ACC progression and suggest that receptor trafficking could be a viable therapeutic target in this malignancy.

**Supplementary Information:**

The online version contains supplementary material available at 10.1186/s12967-025-07146-2.

## Introduction

ACC is a rare and aggressive endocrine malignancy characterized by poor prognosis, frequent metastasis, and resistance to conventional therapies [1]. Although surgical resection remains the primary treatment for localized disease, recurrence and progression are common in advanced-stage patients, with limited effective systemic options available [2]. The complex molecular landscape of ACC poses a significant barrier to the development of targeted therapies, highlighting the need for identification of novel biomarkers and mechanistic insights into ACC pathogenesis.

SorCS3 is a type I transmembrane protein that belongs to the VPS10P domain receptor family, primarily known for its roles in the nervous system [3, 4]. It participates in intracellular trafficking, synaptic plasticity, and receptor recycling [5–7]. Previous studies have revealed that SorCS3 negatively regulates BDNF signaling and modulates anxiety-like behaviors through TrkB internalization in neurons [8, 9]. In GBM, SorCS3 has been reported to suppress tumor proliferation and invasion by modulating the p75NTR signaling pathway [10]. Furthermore, High expression of SorCS3 as a signature was associated with poor prognosis and recurrent disease in GC [11]. These findings suggest that SorCS3 may exert context-dependent tumor suppressive functions beyond the nervous system, yet its role in adrenal cortical tumorigenesis has not been previously characterized.

In our analysis of TCGA, SorCS3 was significantly downregulated in ACC tissues compared to normal adrenal samples, and its low expression correlated with poor overall and disease-free survival. These findings were validated at the mRNA and protein levels in clinical ACC samples and cell lines, suggesting a potential tumor-suppressive role for SorCS3 in this malignancy.

The IGF2R, also known as the cation-independent mannose-6-phosphate receptor, is a multifunctional trafficking receptor involved in lysosomal enzyme targeting, growth factor clearance, and TGF-β activation [12]. Unlike the insulin-like growth factor 1 receptor (IGF1R) [13], IGF2R lacks intrinsic kinase activity and is widely considered a tumor suppressor due to its ability to bind and degrade IGF2, a potent mitogen frequently overexpressed in multiple cancers including ACC [14–16]. Loss of IGF2R expression or function leads to sustained IGF2 signaling and contributes to tumor progression, invasion, and angiogenesis via the PI3K/Akt and MAPK/Erk pathways [17, 18]. However, IGF2R can also exhibit context-dependent pro-tumorigenic roles, particularly through interactions with intracellular trafficking machinery [19] and regulation of cell surface receptor recycling [20], which remain poorly defined in ACC. Preliminary studies have suggested that alterations in SorCS3 may be associated with cancer pathogenesis through mechanisms such as dysregulated cell signaling and impaired cellular trafficking [10]. Given the complex interplay of genetic factors in ACC [21], understanding the specific contributions of SorCS3 could provide valuable insights into the molecular landscape of this malignancy.

In this study, we demonstrate that SorCS3 physically interacts with IGF2R and promotes its stabilization and internalization, suggesting a novel mechanism through which SorCS3 may influence receptor trafficking and signal attenuation in ACC cells. Functional assays revealed that OE-SorCS3 inhibited ACC cell proliferation, migration, and survival, accompanied by downregulation of the PI3K/Akt and MAPK/Erk signaling pathways. These effects were reversed upon pharmacologic inhibition of endocytosis, indicating that SorCS3 exerts its tumor-suppressive functions in an endocytosis dependent manner.

Collectively, our findings identify SorCS3 as a novel tumor suppressor in ACC and uncover its functional interaction with IGF2R as a critical axis regulating intracellular signaling and tumor cell behavior. These results provide new mechanistic insights into the pathogenesis of ACC and suggest that targeting the SorCS3-IGF2R pathway may represent a promising therapeutic strategy for patients with this challenging malignancy.

## Materials and methods

### Cell culture and human ACC discard samples

ACC cell lines SW-13 and NCI-H295R were purchased from Wuhan Prichella Biotechnology Co. Ltd. These cells were cultured in DMEM medium (HyClone, SH3002201) containing 10% fetal bovine serum (Biological Industries, 04–001-1A) and 1% (vol/vol) penicillin/streptomycin (Beyotime, C0222) in an incubator (37 °C, 5% CO_2_).

Patient-derived discarded tissues were obtained from the Department of General Surgery, Jiaxing First Hospital, following relevant guidelines and after obtaining informed consent and institutional review board approval (LS2021-KY-006).

### Public data mining and survival analyses

TCGA-ACC tumor RNA-seq expression data used in this study were retrieved via Home for Researchers (https://www.home-for-researchers.com) and the GEPIA web server (http://gepia.cancer-pku.cn) on May 5, 2022. For comparisons with normal tissues, normal adrenal expression data were obtained from GTEx as implemented in GEPIA, accessed on May 5, 2022. The underlying data originate from TCGA and GTEx.

Patients were dichotomized into SorCS3-high and SorCS3-low cohorts by median (or by maximally selected rank statistics where specified). OS and disease-free survival (DFS) were evaluated by Kaplan–Meier analysis with log-rank tests; hazard ratios (HRs) with 95% CIs were estimated using Cox proportional hazards models (univariate and multivariate where indicated). Time-dependent ROC curves were computed with the timeROC package to estimate AUC at 1-, 3-, and 5-year time points. Stage-stratified analyses (TNM) and subgroup analyses by radiotherapy status were pre-specified. Multiple testing for transcriptome-scale comparisons used Benjamini–Hochberg FDR control.

### Transient overexpression of SorCS3

The full-length human SorCS3 ORF (RefSeq NM_014979) was cloned into pcDNA3.1(+) for transient expression, with a C-terminal 3 × FLAG tag. All constructs were verified by Sanger sequencing, and endotoxin-free plasmid preparations were used.

Transfection procedure (Lipofectamine 3000). DNA–lipid complexes were prepared in Opti-MEM according to the manufacturer’s protocol. For a 6-well format, 2.5 µg pcDNA3.1-SorCS3 was mixed with 2 µL P3000 in 125 µL Opti-MEM; in a separate tube, 3.75 µL Lipofectamine 3000 was diluted in 125 µL Opti-MEM. After 10–15 min at room temperature, the mixtures were combined and incubated for 10 min, then added dropwise to cells in complete medium. Medium was refreshed after 6 h.

### ASO-mediated knockdown of SorCS3

A chemically synthesized antisense oligonucleotide targeting SorCS3 (ASO-SorCS3; sequence 5′-CUUGGGATGAAAGAGCAGGC-3′) and a non-targeting control ASO were transfected using Lipofectamine RNAiMAX (Thermo Fisher). For a 6-well format, complexes were prepared in Opti-MEM as follows: dilute 100 pmol ASO (to yield a 50 nM final concentration in 2 mL/well) in 125 µL Opti-MEM; in a separate tube dilute 4 µL RNAiMAX in 125 µL Opti-MEM. Incubate each for 10–15 min, combine, incubate 10 min, and add dropwise to cells in antibiotic-free complete medium. Replace medium after 6–8 h. Cells were harvested 24–48 h post-transfection for RNA and 48–72 h for protein and functional assays. Knockdown efficiency was confirmed by qPCR and Western blot.

### Transwell migration and invasion assays

NCI-H295R and SW-13 cells overexpressing SorCS3 (as described above) were serum-starved (0.5% FBS, 6–12 h) before assays. For migration, 24-well Transwell inserts (8-µm pores) were used; 5 × 10^4 (NCI-H295R) or 4 × 10^4 (SW-13) cells in 100–150 µL serum-reduced medium were seeded in the upper chamber, with 600 µL complete medium (10% FBS) in the lower chamber, and incubated 12–16 h. For invasion, inserts were pre-coated with growth-factor-reduced Matrigel (1:8, 50 µL/insert, 30–45 min, 37 °C). 5 × 10^5 (NCI-H295R) or 2 × 10^5 (SW-13) cells were seeded in serum-free/0.5% FBS medium; the lower chamber contained 10% FBS medium; incubation was 20–24 h. Cells received Dynasore (40 µM) or vehicle (0.1% DMSO) with a 120 min pretreatment prior to seeding; the same concentration was maintained in both chambers for the assay duration. After incubation, non-migrated/non-invaded cells were removed from the upper membrane surface. The membranes were fixed (4% PFA, 15 min) and stained (0.1% crystal violet, 30 min). Five random fields per insert were imaged at 200×, and cells were counted by a blinded operator.

### Cell-cycle analysis by flow cytometry

Cells were harvested at ~ 70–80% confluence, washed twice with ice-cold PBS, and fixed in pre-chilled 75% ethanol at − 20 °C overnight. Fixed cells were washed twice with PBS to remove ethanol and incubated in RNase A (100 µg/mL, 30 min, 37 °C) followed by propidium iodide (PI, 50 µg/mL) for 15 min at room temperature, protected from light. Data were acquired on a benchtop flow cytometer (BD Biosciences) with linear area detection for PI. At least 10,000 single-cell events were collected per sample. Biological replicates (n ≥ 3) were processed in parallel.

### Apoptosis analysis by annexin V–FITC/PI staining

Cells were trypsinized without EDTA, pooled with floating cells, and washed twice with cold PBS. 5 × 10^5 cells were resuspended in binding buffer, incubated with Annexin V–FITC for 10 min in the dark, followed by PI (5 µL; 50 µg/mL) for 5 min, then brought to 300–500 µL with binding buffer. At least 10,000 singlet events were recorded per sample; gating was applied uniformly across replicates (n ≥ 3).

### Molecular docking and structural visualization

Protein–protein docking between SorCS3 and IGF2R was performed on the WeMol web platform with default parameters unless specified (WeMol, Wecomput Technology Co., Ltd, ttps://wemol.wecomput.com). Briefly, input structures for the extracellular regions were prepared by removing signal peptides, transmembrane helices, heteroatoms, and alternate conformations; missing side chains were completed and protonation states assigned at pH 7.4 during preprocessing. The WeMol protein–protein docking module generated and ranked candidate complexes; top-ranked poses were filtered by docking score and cluster size, and steric plausibility was verified by visual inspection. Three independent runs yielded consistent top clusters. Interface analysis (buried surface area, hydrogen bonds/contacts, and distance measurements) and figure rendering were carried out in UCSF ChimeraX (v1.6). Publication images used ribbon representation with surfaces at the interface; residues at putative contact patches were labeled for clarity, and distances/hydrogen bonds were annotated using built-in ChimeraX tools.

### Western blot and band densitometry

Total protein was extracted using RIPA buffer and protein expression was analyzed by Western blotting as described previously. GAPDH served as an endogenous control. The antibodies information utilized in Western blotting has been listed in Supplementary File 1: Table S1. Chemiluminescent blots were imaged as 16-bit TIFF files without gamma adjustment using a digital imager, with exposure times chosen to avoid saturation. Band intensities were quantified in ImageJ using the Gel Analyzer workflow. Rectangular ROIs of identical size were placed over each band; local background was removed, and Integrated Density was recorded.

### Immunohistochemistry (IHC)

FFPE sections of ACC and adjacent adrenal tissues (4 µm) were deparaffinized, rehydrated, and subjected to heat-induced epitope retrieval (10 mM sodium citrate, pH 6.0, 15–20 min). Endogenous peroxidase was quenched with 3% H_2_O_2_ (10 min). After blocking, slides were incubated with primary antibodies against SorCS3 and IGF2R (dilutions/sources in Table S1) overnight at 4 °C, followed by an HRP-polymer secondary (30 min, RT), DAB development, hematoxylin counterstain, dehydration, and mounting. Negative controls (primary antibody omitted) were included in each run. Staining was semi-quantified by H-score (0–300) by two blinded reviewers; discrepancies were resolved by consensus. Images were acquired under identical exposure settings.

#### 5-FAM, SE-BSA internalization assay

5-FAM, SE (Invitrogen, C2210) is a fluorescent labelling reagent that exists as a single isomer to bind peptides, proteins and nucleotides via chemical reactions. According to the manufacturer’s protocol, fluorescently labelled bovine serum albumin (BSA, Sigma, A1933) was prepared by incubation in the dark. 5-FAM, SE-BSA collected from the cut-off column was filtered through a PVDF filter to remove bacteria. 5-FAM, SE-BSA was added to cultured glioma cells to observe the internalization ability under a fluorescence microscope.

#### Fluorescence quantification and statistics

Images were acquired under identical microscope settings (objective, exposure, gain, laser power) within each experiment while avoiding saturation. For each well, ≥ 4 fields were collected at random positions. Nuclei were counterstained with DAPI/Hoechst and segmented by adaptive thresholding with watershed separation to obtain the nuclei count per field. Background for the 5-FAM channel was estimated from ≥ 3 cell-free regions in the same well and subtracted from each image prior to quantification. The 5-FAM signal was summarized as Integrated Density (background-corrected intensity summed over the field). Field-level fluorescence was then normalized to cell number as:

Normalized value = (Integrated density of 5-FAM)/(Number of Nuclei).

#### Nude mice tumorigenicity assay

Our study was approved by the Ethics Committee of Harbin Medical University (Daqing). Nude mice were randomly grouped into the control group and the OE-SorCS3 group. Mice were inoculated subcutaneously in the right flanks with 1 × 10^7 SW-13 cells transfected with the negative control vector or the LV-EFS > Kozak-SorCS3-CMV > EGFP/T2A/Puro.

#### Co-immunoprecipitation

Co-IP was performed using a Pierce Co-IP Kit (Thermo Scientific) according to the manufacturer’s protocol. According to experimental needs, a purified anti-Flag antibody was used to IP the antigen, and any co-immunoprecipitated interacting proteins were immobilized directly onto an amine-reactive resin by covalent coupling. The final protein eluate was denatured and analysed by western blotting.

#### Statistical analysis

Data are presented as mean ± SEM. Significance was determined by using Student’s t-test for comparison of two groups. All statistical tests were performed using Prism. Differences with *P* < 0.05 were considered significant (*P < 0.05, **P < 0.01, ***P < 0.001). Analyses were conducted in GraphPad Prism v9.5 (GraphPad Software, San Diego, CA, USA).

## Results

### SorCS3 is downregulated in ACC and correlates with poor prognosis

**Fig. 1:**
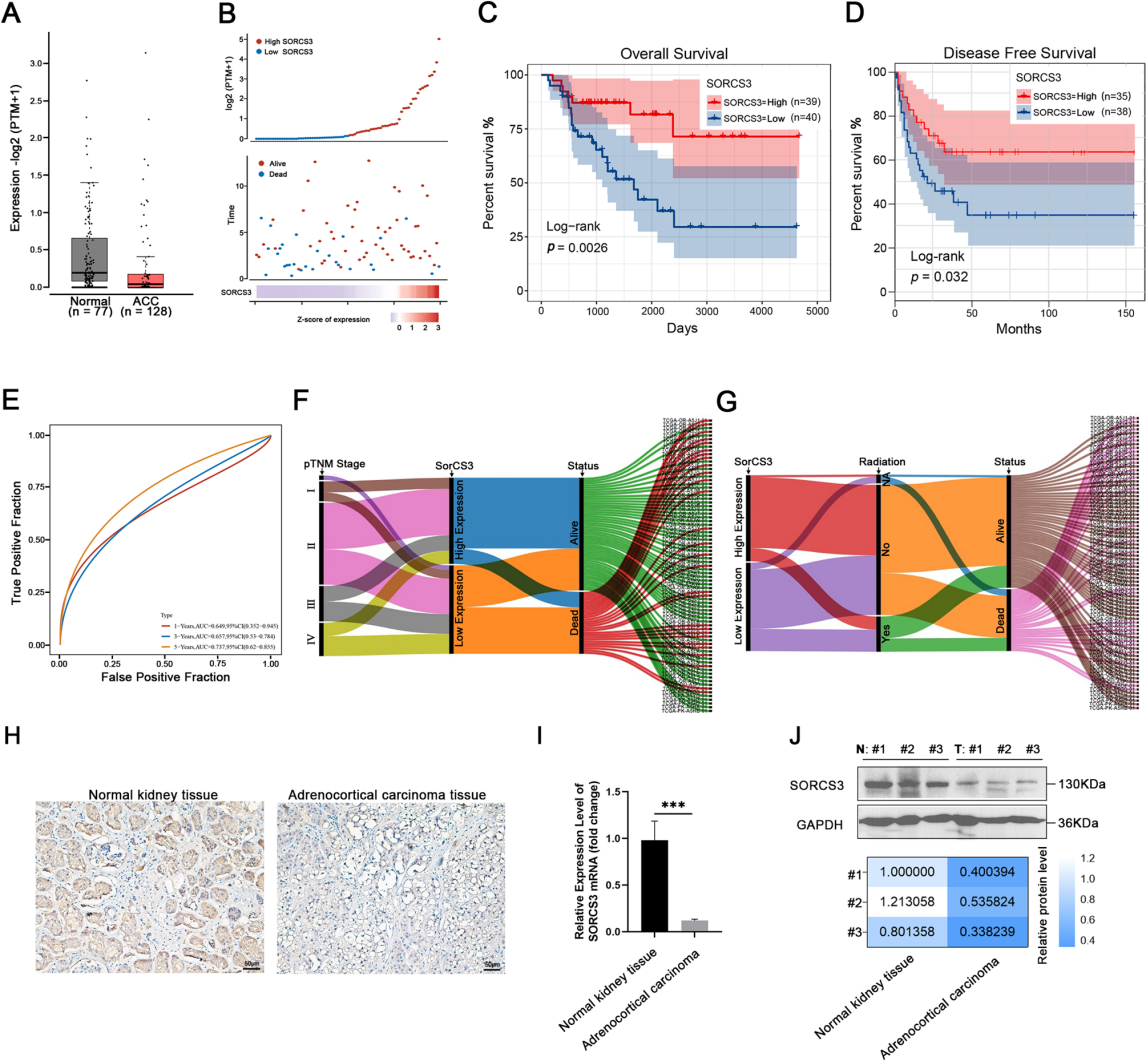
Downregulation of SorCS3 is associated with poor prognosis in ACC. **A** Expression levels of SorCS3 in normal tissues (n = 77) and ACC tissues (n = 128) from TCGA datasets, presented as log_2_ TPM. **B** Expression distribution of SorCS3 among ACC patients, grouped by survival status. **C** Kaplan–Meier analysis of OS in patients with high (n = 39) and low (n = 40) SorCS3 expression. **D** Kaplan–Meier analysis of disease-free survival (DFS) in high (n = 35) and low (n = 38) SorCS3 expression groups. **E** ROC curve analysis evaluating the predictive accuracy of SorCS3 expression for 1, 3, and 5-year survival in ACC. **F** Sankey diagram showing relationships among pTNM stage, SorCS3 expression, and patient survival status. **G** Sankey diagram showing relationships among SorCS3 expression, radiation treatment, and survival status. **H** Representative IHC images of SorCS3 protein expression in normal kidney tissue and adrenocortical carcinoma tissue. Scale bar = 50 μm. **I** qRT-PCR analysis of SorCS3 mRNA expression in three paired normal kidney and ACC tissues (***P < 0.001). **J** Western blot analysis of SorCS3 expression in three paired normal kidney and ACC tissues. GAPDH served as the internal control. Densitometric analysis is shown as relative protein levels

To investigate the expression pattern of SorCS3 in ACC, we analyzed transcriptomic data from TCGA. The results revealed that SorCS3 expression was significantly downregulated in ACC tissues compared to normal adrenal tissues (Fig. 1A). Kaplan–Meier survival analysis demonstrated that ACC patients with low SorCS3 expression exhibited significantly shorter OS and DFS compared to those with high SorCS3 expression (Fig. 1B–D). Furthermore, time-dependent receiver operating characteristic (ROC) curve analysis showed that SorCS3 expression had a strong predictive value for patient survival, as indicated by high area under the curve (AUC) values at various time points (Fig. 1E). Stratification by TNM stage indicated that SorCS3-high expression was more frequently observed in early-stage ACC, while patients with advanced-stage disease more commonly exhibited low SorCS3 expression (Fig. 1F). Notably, patients with high SorCS3 expression showed favorable prognosis even in the absence of radiotherapy, further supporting its potential protective role (Fig. 1G). To validate these findings, we performed IHC staining on three clinical ACC samples (Supplementary Fig. 1A), which confirmed markedly decreased SorCS3 protein expression in tumor tissues compared to adjacent normal adrenal tissues. Consistently, SorCS3 mRNA and protein levels were both significantly reduced in ACC samples (Fig. 1H–J and Supplementary Fig. 1B).

Taken together, these findings indicate that SorCS3 is frequently downregulated in ACC and its low expression is associated with poor prognosis, suggesting that SorCS3 may function as a potential tumor suppressor in ACC.

### OE-SorCS3 suppresses proliferation, invasion, and migration of ACC cells

**Fig. 2:**
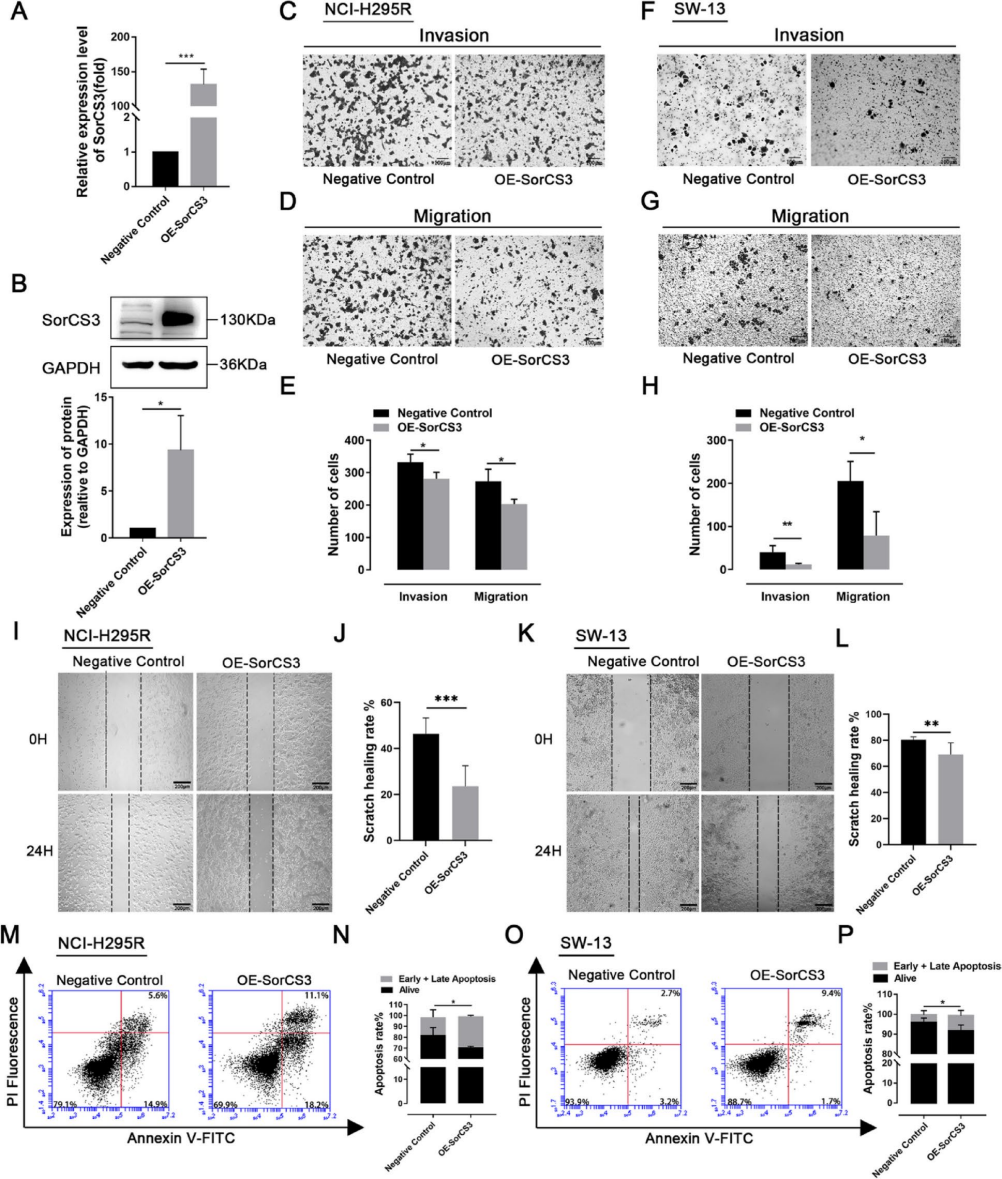
Overexpression of SorCS3 suppresses cell invasion and migration while promoting apoptosis in ACC cell lines. **A** and **B** After transfection with the pCMV3-SorCS3-Flag plasmid, the expression levels of SorCS3 were examined through real-time PCR and Western blotting, using GAPDH as an endogenous control. **C**–**E** Transwell assays in NCI-H295R cells show reduced invasion and migration upon SorCS3 overexpression; quantification shown in (**E**). **F**–**H** Transwell assays in SW-13 cells show reduced invasion and migration upon SorCS3 overexpression; quantification shown in (**H**). **I** and **L** Wound-healing assay in NCI-H295R(I) and SW13(K) cells demonstrates accelerated scratch closure following SorCS3 overexpression; quantification shown to the right (**J** and **L**). **M**–**P** Flow cytometry was used to detect apoptosis in NCI-H295R (**M**) and SW-13 (**O**) cells, quantification shown in (**N** and **P**). Data are presented as mean ± SD; *P < 0.05, **P < 0.01, ***P < 0.001

**Fig. 3:**
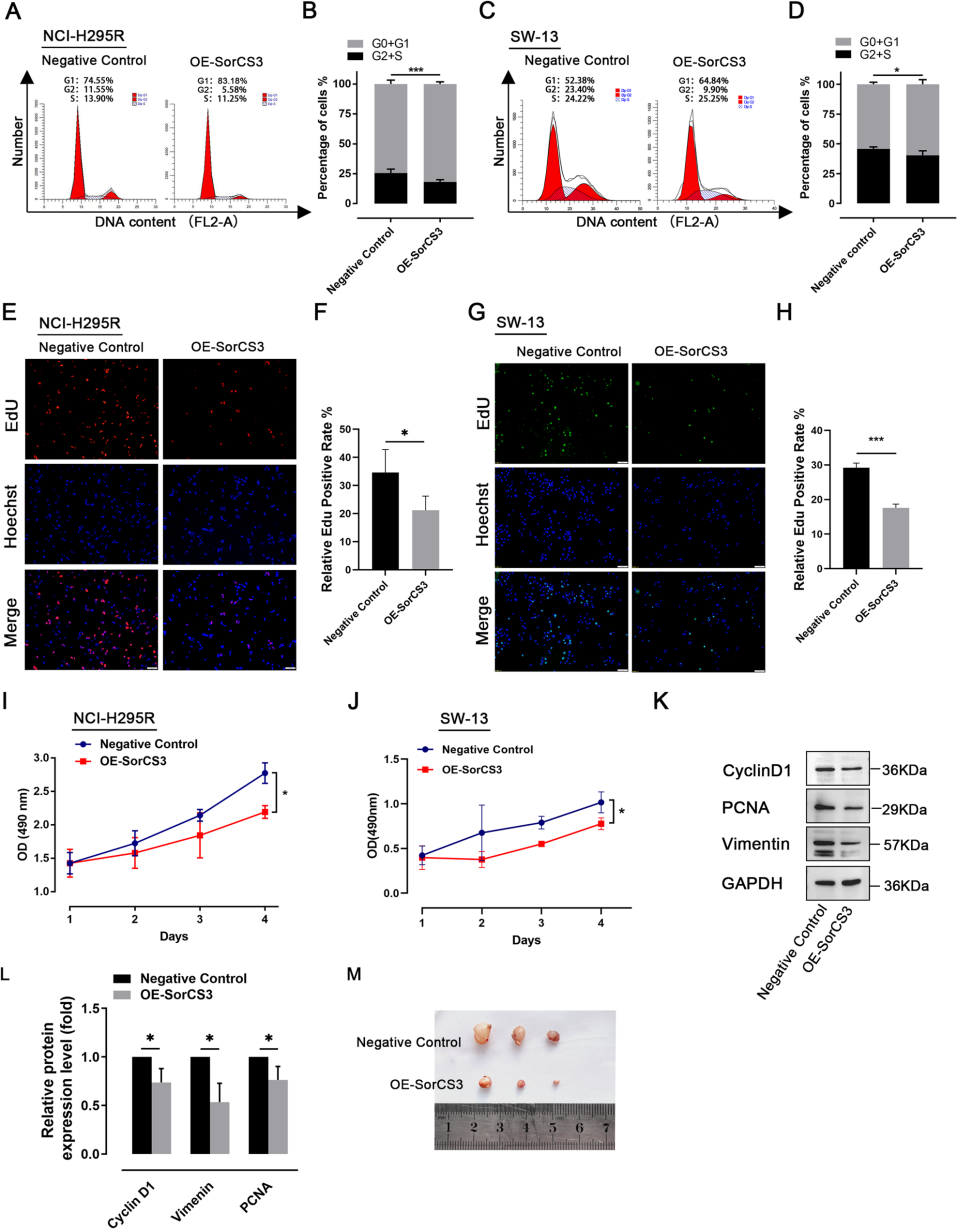
SorCS3 overexpression induces cell-cycle arrest and suppresses proliferation of adrenocortical carcinoma cells in vitro and in vivo. **A**–**B** Flow-cytometry analysis of cell-cycle distribution in NCI-H295R cells shows an increased G0/G1 fraction and a reduced S phase upon SorCS3 overexpression; quantification shown in (**B**). **C** and **D** Similar G0/G1 arrest is observed in SW-13 cells; quantification shown in (**D**). **E**–**F** EdU assay, NCI-H295R: EdU-positive nuclei (red) and Hoechst (blue); color legend is indicated on each panel. The EdU-positive rate (%) = EdU⁺/ Hoechst⁺ × 100%; quantification shown in (**F**). **G**–**H** EdU assay, SW-13: EdU-positive nuclei (green) and Hoechst (blue) with the same palette and legend as in (**E**); quantification shown in (**H**). **I**–**J** CCK-8 assays show reduced cell viability over time in NCI-H295R (**I**) and SW-13 (**J**) upon SorCS3 overexpression. **K**–**L** WB of cell-cycle and mesenchymal markers in NCI-H295R cells. SorCS3 overexpression decreases Cyclin D1, PCNA, and Vimentin protein levels (**K**); densitometric quantification normalized to GAPDH and to the Negative Control is shown in (**L**). **M** Representative xenograft tumors derived from NCI-H295R cells in nude mice show smaller tumor size in the SorCS3-overexpression group than in the negative control. Data are mean ± SD from ≥ 3 independent experiments. *P < 0.05, **P < 0.01, ***P < 0.001

To investigate the biological function of SorCS3 in ACC, we established OE-SorCS3 models in two ACC cell lines, NCI-H295R and SW-13. qPCR and Western blot analyses confirmed a significant upregulation of SorCS3 expression in the transfected cells (Fig. 2A, B). In Transwell assays, ectopic SorCS3 significantly reduced the numbers of cells crossing the membrane in both NCI-H295R and SW-13, indicating diminished invasive and migratory capacities (Fig. 2C–H). Consistently, wound-healing assays showed a slower scratch-closure rate upon OE-SorCS3 in the two cell lines (Fig. 2I–L). Together, these data demonstrate that SorCS3 restrains ACC cell motility and invasiveness in vitro. Western blotting confirmed efficient SorCS3 depletion by ASO-SorCS3 (Supplementary Fig. 2A, B). In Transwell assays, SorCS3 knockdown increased the number of NCI-H295R and SW-13 cells traversing the membrane, indicating enhanced invasive and migratory capacities (Supplementary Fig. 2C–F). Consistently, wound-healing assays showed an accelerated scratch-closure rate upon SorCS3 loss in both lines (Supplementary Fig. 2G, H). These loss-of-function data phenocopy the inverse of the overexpression results and further support a motility-suppressive role of SorCS3 in ACC cells. Further analysis of apoptosis using flow cytometry revealed a significantly increased proportion of early and late apoptotic cells in the OE-SorCS3 group compared to the control group (Fig. 2M–P), indicating that SorCS3 promotes apoptosis in ACC cells. Cell cycle analysis showed that OE-SorCS3 induced a marked G0/G1 phase arrest, with a corresponding reduction in S-phase cell population (Fig. 3A–D). Additionally, EdU assays confirmed decreased DNA synthesis activity in OE-SorCS3 cells (Fig. 3E–H and Supplementary Fig. 2I–J). CCK-8 assays demonstrated that OE-SorCS3 significantly inhibited the proliferation of NCI-H295R and SW-13 cells in vitro (Fig. 3I–J). We next examined the expression of key markers associated with cell proliferation and migration. Notably, OE-SorCS3 resulted in a marked reduction in the protein levels of PCNA, Cyclin D1, and Vimentin (Fig. 3K, L). Consistent with these findings, subcutaneous xenograft models in mice showed that tumors formed in the OE-SorCS3 group were significantly smaller in volume compared to those in the control group (Fig. 3M), further supporting the in vivo tumor suppressive effect of SorCS3.

Taken together, these results indicate that SorCS3 exerts potent tumor-suppressive functions in ACC by inducing apoptosis, causing cell cycle arrest, and inhibiting cell invasion, migration, and proliferation, highlighting its potential as a therapeutic target in ACC.

### SorCS3 has an internalization function in ACC

**Fig. 4:**
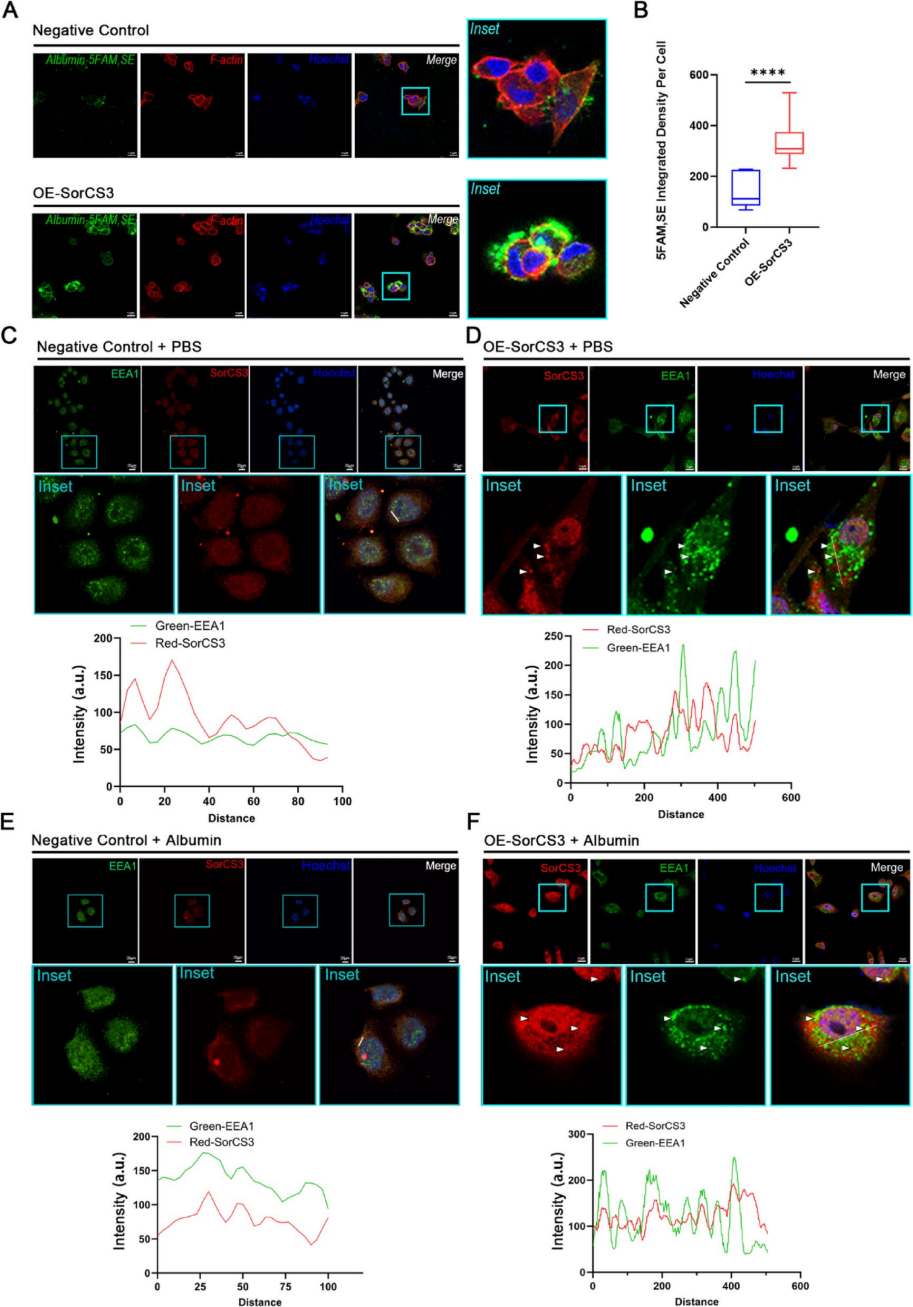
SorCS3 regulates specific ligand internalization in ACC. **A** Representative confocal images showing albumin (green), F-actin (red), and nuclei (blue) in negative control and OE-SorCS3 NCI-H295R cells. Scale bar = 17 μm; inset magnification on the right. **B** Quantification of relative fluorescence intensity (Integrated Density of 5-FAM)/(Number of Nuclei), showing significantly increased albumin uptake in OE-SorCS3 cells (****P < 0.0001). **C** and **D** Confocal images of Negative Control (**C**) and OE-SorCS3 (**D**) cells treated with PBS, showing partial colocalization of SorCS3 (red) with early endosome marker EEA1 (green); nuclei stained with Hoechst (blue). Inset shows higher magnification of colocalized puncta (arrowheads). Line-scan analysis of fluorescence intensity along a selected region from (**C**–**D**), demonstrating low overlap between SorCS3 and EEA1 signals. **E**–**F** Confocal images of Negative Control (**E**) and OE-SorCS3 (**F**) cells treated with albumin showing increased colocalization of SorCS3 (red) with EEA1 (green). Insets highlight more frequent colocalized puncta. Line-scan analysis from (**E**–**F**) reveals enhanced colocalization between SorCS3 and EEA1 following albumin stimulation. a.u., arbitrary units. Data are presented as mean ± SD

To explore the biological role of SorCS3 in ACC cells, we performed Albumin-5, FAM uptake assay and colocalization analysis. In OE-SorCS3 cells, we observed a significant increase in the intracellular accumulation of fluorescently labeled albumin compared with the negative control, which was observed by confocal microscopy (Fig. 4A). Fluorescence intensity quantitative analysis showed that albumin uptake was significantly increased in OE-SorCS3 cells (Fig. 4B), indicating enhanced internalization. To further evaluate the endosomal localization of SorCS3, we detected its colocalization with the early endosomal marker EEA1. In the PBS-treated OE-SorCS3 group, partial colocalization of SorCS3 and EEA1 was detected (Fig. 4C, D), while albumin stimulation significantly enhanced this colocalization, which was confirmed by the increase in overlapping fluorescence signals and intensity distribution analysis (Fig. 4E, F). These results indicate that SorCS3 is involved in internalization trafficking and may promote cargo delivery to early endosomes.

Taken together, these results suggest that SorCS3 has internalization capacity in ACC cells and may play a role in mediating the intracellular trafficking of specific ligands.

### SorCS3 interacts with IGF2R and regulates its expression in ACC

**Fig. 5:**
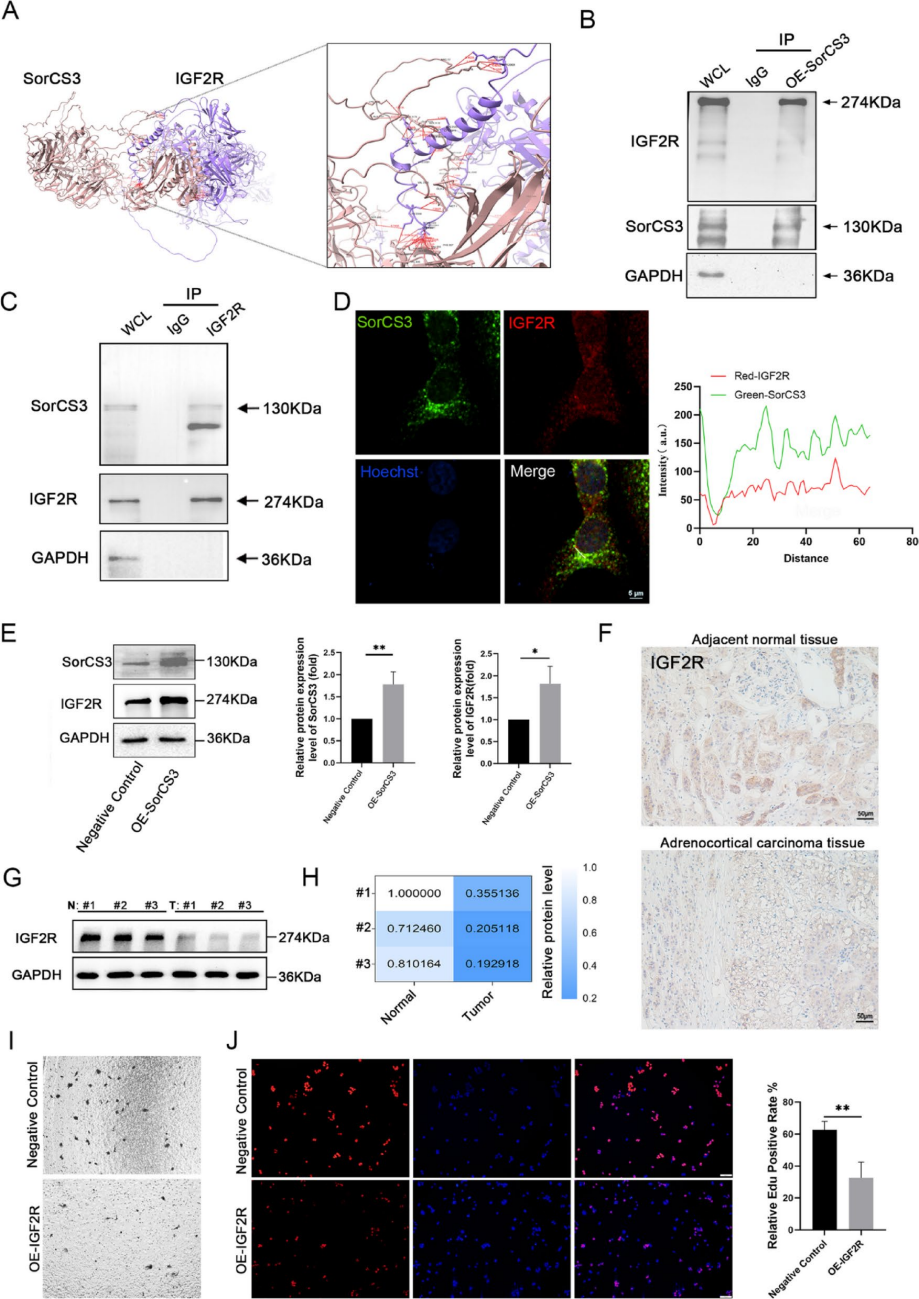
SorCS3 interacts with and upregulates IGF2R expression in ACC. **A** Predicted structural model of the interaction between SorCS3 (brown) and IGF2R (purple), with a zoomed-in view showing the predicted binding interface. **B** Co-IP assay demonstrating the interaction between overexpressed SorCS3 and endogenous IGF2R in ACC cells. IgG and whole cell lysate (WCL) served as controls. **C** Co-IP assay demonstrating the interaction between endogenous SorCS3 and endogenous IGF2R in ACC cells. IgG and WCL served as controls. **D** Immunofluorescence staining of SorCS3 (green), IGF2R (red), and nuclei (Hoechst, blue) in ACC cells. Merged images show subcellular co-localization of SorCS3 and IGF2R. Scale bar = 5 μm. Fluorescence intensity profile along the line drawn in (**D**), showing overlapping signal peaks of SorCS3 and IGF2R. **E** Western blot analysis showing increased expression of both SorCS3 and IGF2R in OE-SorCS3 NCI-H295R cells compared to negative control. GAPDH was used as the internal loading control. **F** Representative IHC images of IGF2R expression in adjacent normal adrenal tissue and ACC tumor tissue, showing reduced IGF2R expression in tumors. Scale bar = 50 μm. **G** and **H** Quantification of SorCS3 (**F**) and IGF2R (**G**) protein expression levels in paired normal kidney and ACC tissues. **I** Transwell invasion assay comparing negative control and OE-IGF2R ACC cells. **J** EdU incorporation assay showing decreased proliferation in OE-IGF2R cells compared to control. Quantification of EdU-positive cells is shown. Data are presented as mean ± SD; *P < 0.05, **P < 0.01

To further investigate the molecular mechanisms underlying the tumor-suppressive function of SorCS3 in ACC, we explored potential interacting proteins. In our previous study, mass spectrometry analysis following Co-IP/MS identified IGF2R as a potential binding partner of SorCS3. Structural modeling suggested a direct protein–protein interaction interface between SorCS3 and IGF2R (Fig. 5A). Co-IP assays in ACC cells confirmed the interaction between SorCS3 and IGF2R. In the OE-SorCS3 group, IGF2R was efficiently pulled down by the anti-SorCS3 antibody (Fig. 5B). Moreover, analysis of endogenous protein complexes further validated this interaction, as SorCS3 was successfully co-precipitated using an anti-IGF2R antibody (Fig. 5C). Furthermore, immunofluorescence analysis revealed a clear co-localization of SorCS3 and IGF2R within ACC cells (Fig. 5D and Supplementary Fig. 5). Western blot analysis demonstrated that OE-SorCS3 significantly increased IGF2R protein levels in ACC cells (Fig. 5E), suggesting that SorCS3 may stabilize or positively regulate IGF2R expression. Consistent with these in vitro findings, IHC staining of clinical samples showed markedly lower IGF2R expression in ACC tissues compared to adjacent normal tissues (Fig. 5F).

To evaluate the clinical significance of IGF2R in ACC, we analyzed patient survival data. Although the differences were not statistically significant, patients with higher IGF2R expression exhibited a trend toward shorter disease-free and overall survival compared to those with lower expression levels (Supplementary Fig. 3A–C). In addition, Western blot analysis of three paired ACC and adjacent normal adrenal tissues confirmed a significant downregulation of IGF2R in tumor tissues (Fig. 5G, H). Functional assays further demonstrated that IGF2R overexpression in ACC cells significantly inhibited both migration and proliferation (Fig. 5I, J), supporting a potential role for IGF2R in modulating tumor behavior. Previous studies have shown that loss of IGF2R expression can result in sustained activation of IGF2 signaling [17, 22]. Accordingly, we also examined IGF2 expression in ACC tissues (Supplementary Fig. 4A, B), and found that OE-SorCS3 altered IGF2 expression levels (Supplementary Fig. 4C, D).

Collectively, these findings indicate that SorCS3 physically interacts with IGF2R and modulates its expression, potentially stabilizing IGF2R in ACC cells. The regulatory effect on IGF2R may contribute, at least in part, to the tumor-suppressive role of SorCS3, and highlights IGF2R as a candidate biomarker and potential therapeutic target in ACC.

### The endocytic inhibitor dynasore reverses the tumor-suppressive effects of SorCS3 in ACC cells

**Fig. 6:**
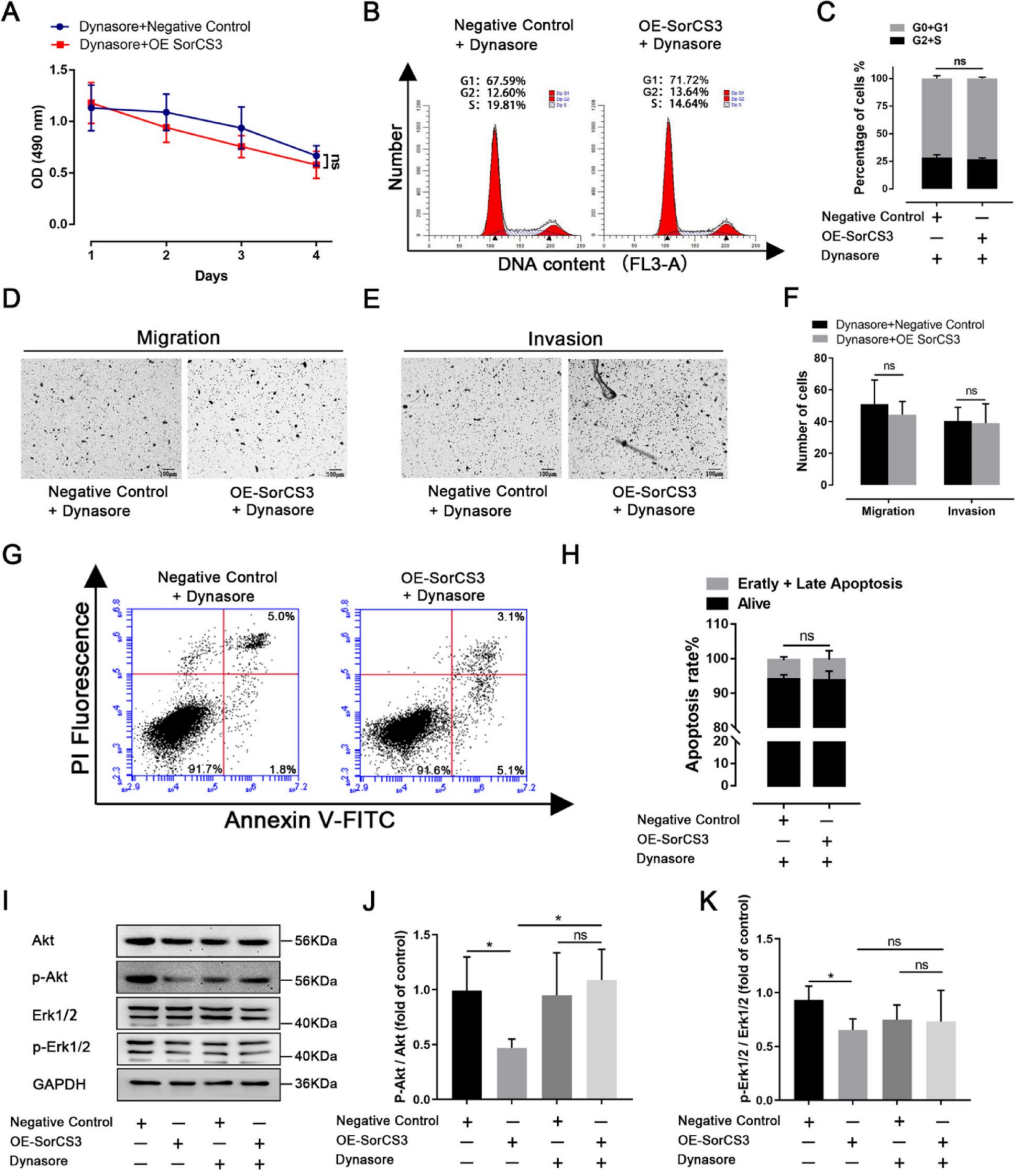
Inhibition of endocytosis by Dynasore attenuates the tumor-suppressive effects of SorCS3 in ACC cells. **A** CCK-8 assay showing reduced cell proliferation in OE-SorCS3 cells, which is partially reversed upon Dynasore treatment. **B** and **C** Flow cytometry analysis of cell cycle distribution in Dynasore treated NCI-H295R cells with or without SorCS3 overexpression. No significant difference in G0/G1 or S phase proportions was observed; quantification shown in (**C**). **D**–**F** Transwell migration (**D**) and invasion (**E**) assays in NCI-H295R cells indicate that Dynasore abrogates SorCS3-overexpression–mediated suppression; quantification is shown in (**F**). **G**–**H** Annexin V-FITC/PI apoptosis assay shows reduced apoptosis in OE-SorCS3 cells upon Dynasore treatment; quantified in (**H**), indicating no significant difference. **I** Western blot analysis of Akt and Erk1/2 signaling pathways. SorCS3 overexpression reduced phosphorylation of Akt and Erk1/2, which was rescued by Dynasore. GAPDH was used as loading control. **J**–**K** Quantification of phosphorylated Akt/total Akt(J) and phosphorylated Erk1/2/total Erk1/2 (**K**) ratios, demonstrating Dynasore reversed the suppressive effect of SorCS3 on Akt and Erk signaling. Data are presented as mean ± SD; *P < 0.05; ns, not significant

To further investigate whether the tumor suppressive effects of SorCS3 are dependent on endocytic processes, we treated ACC cells with Dynasore, a specific inhibitor of dynamin-dependent endocytosis. CCK-8 assays demonstrated that OE-SorCS3 cells treated with Dynasore exhibited no significant difference in cell proliferation compared to control cells, suggesting that Dynasore effectively blocked SorCS3-mediated growth inhibition (Fig. 6A). Flow cytometry analysis revealed that Dynasore treatment abolished the SorCS3-induced G1 phase arrest, as no significant differences in the distribution of cell cycle phases were observed between OE-SorCS3 and control groups under Dynasore treatment (Fig. 6B, C). Consistently, transwell migration and invasion assays showed that the inhibitory effects of SorCS3 on cell migration and invasion were also diminished in the presence of Dynasore (Fig. 6D, F and Supplementary Fig. 6). Moreover, Annexin V/PI staining showed no significant increase in apoptosis in OE-SorCS3 cells treated with Dynasore compared to control cells (Fig. 6G, H), suggesting that Dynasore reverses SorCS3-mediated pro-apoptotic effects. Western blot analysis further revealed that OE-SorCS3 significantly suppressed the phosphorylation of Akt and Erk1/2; however, this inhibitory effect was abolished by Dynasore treatment (Fig. 6I–K). These results indicate that SorCS3 suppresses cell proliferation, migration, invasion, and survival through endocytosis-dependent inhibition of the Akt and Erk signaling pathways.

Taken together, these findings suggest that the tumor-suppressive functions of SorCS3 in ACC are mediated, at least in part, through internalization mechanisms involving downregulation of the PI3K/Akt and MAPK/Erk pathways.

## Discussion

ACC is a rare but aggressive malignancy with limited therapeutic options and poor prognosis [23, 24]. In this study, we identified SorCS3, a member of the VPS10p-domain receptor family previously thought to be brain-specific, as a novel tumor suppressor candidate in ACC. We demonstrated that SorCS3 is expressed in both ACC tissues and cell lines, and its low expression was significantly associated with poorer OS and DSS in the TCGA cohort, underscoring its potential clinical relevance (Fig. 1).

SorCS3 is known to regulate receptor trafficking and endocytosis in neurons [25, 26], and our findings extend this function to ACC cells. Specifically, SorCS3 overexpression enhanced endocytic activity and colocalized with early endosomes, as indicated by EEA1 staining (Fig. 4). Importantly, SorCS3 interacts with the IGF2R a multifunctional receptor involved in IGF2 degradation and known to suppress tumor progression by dampening PI3K/Akt and MAPK/Erk signaling pathways. We observed that SorCS3 overexpression increased IGF2R protein levels and concurrently reduced the phosphorylation of Akt and Erk, suggesting suppression of key oncogenic signaling cascades (Figs. 5 and 6). These findings support a model in which SorCS3 stabilizes IGF2R via enhanced endocytic trafficking, contributing to its tumor-suppressive effect in ACC.

A point of complexity is the discrepancy between our findings and previously published data regarding IGF2R expression in ACC [15]. This study demonstrates the presence of a high IGF2R protein expression in most ACCs, suggesting that a high level of IGF2R protein might counteract the growth-stimulating effects of IGF2 in adrenocortical tumorigenesis [15]. In contrast, our analysis of clinical specimens revealed a reduction in IGF2R protein expression, paralleling that of SorCS3 (Fig. 1J and 5F–G). This inconsistency may reflect differences in detection levels (mRNA vs. protein), post-transcriptional regulation, tumor stage, or sample heterogeneity. Receptor protein levels are often influenced by degradation mechanisms, trafficking efficiency, and microenvironmental factors, all of which are highly dynamic in cancer [27, 28]. Moreover, recent studies emphasize the context-dependent dual roles of IGF2R in cancer [29, 30]. While commonly viewed as a tumor suppressor by degrading IGF2 as in hepatocellular [29] and bladder cancers [31] IGF2R has also been implicated in tumor progression. For instance, it can promote breast cancer invasion via αVβ3 integrin processing and mediate immune evasion in lung adenocarcinoma through TGF-β activation [20].

Collectively, our study expands the functional repertoire of SorCS3 beyond the nervous system and highlights endocytic trafficking as a critical regulatory layer of receptor-mediated signaling in ACC. The identification of a potential SorCS3–IGF2R–Akt/Erk axis underscores the importance of vesicular transport in tumor biology and opens new avenues for therapeutic intervention aimed at modulating receptor fate and downstream signaling.

Nonetheless, this study has several limitations. First, the number of clinical ACC tissue samples analyzed was relatively limited, which may affect the statistical power and generalizability of our findings. Second, although our study proposes SorCS3 as a potential therapeutic target in ACC, we have not yet conducted pharmacological evaluations to validate its druggability. The current findings remain at the mechanistic level, without exploration of small-molecule modulators, peptide mimetics, or in vivo efficacy assessments. Future studies should focus on structure-based drug design and targeted delivery strategies to assess the feasibility of translating SorCS3 modulation into clinical applications.

## Supplementary Information

Below is the link to the electronic supplementary material.

Supplementary Material 1.

Supplementary Material 2.

Supplementary Material 3.

Supplementary Material 4.

Supplementary Material 5.

Supplementary Material 6.

Supplementary Material 7.

Supplementary Material 8.

Supplementary Material 9.

Supplementary Material 10.

Supplementary Material 11.

## Data Availability

Data will be made available on request.
