# Supplementary figures and images for: SorCS3 suppresses adrenocortical carcinoma progression by enhancing IGF2R-mediated endocytic trafficking and signaling attenuation
Source: J Transl Med. 2025 Oct 21;23:1146. doi: 10.1186/s12967-025-07146-2 (PMC12538998; doi:10.1186/s12967-025-07146-2)

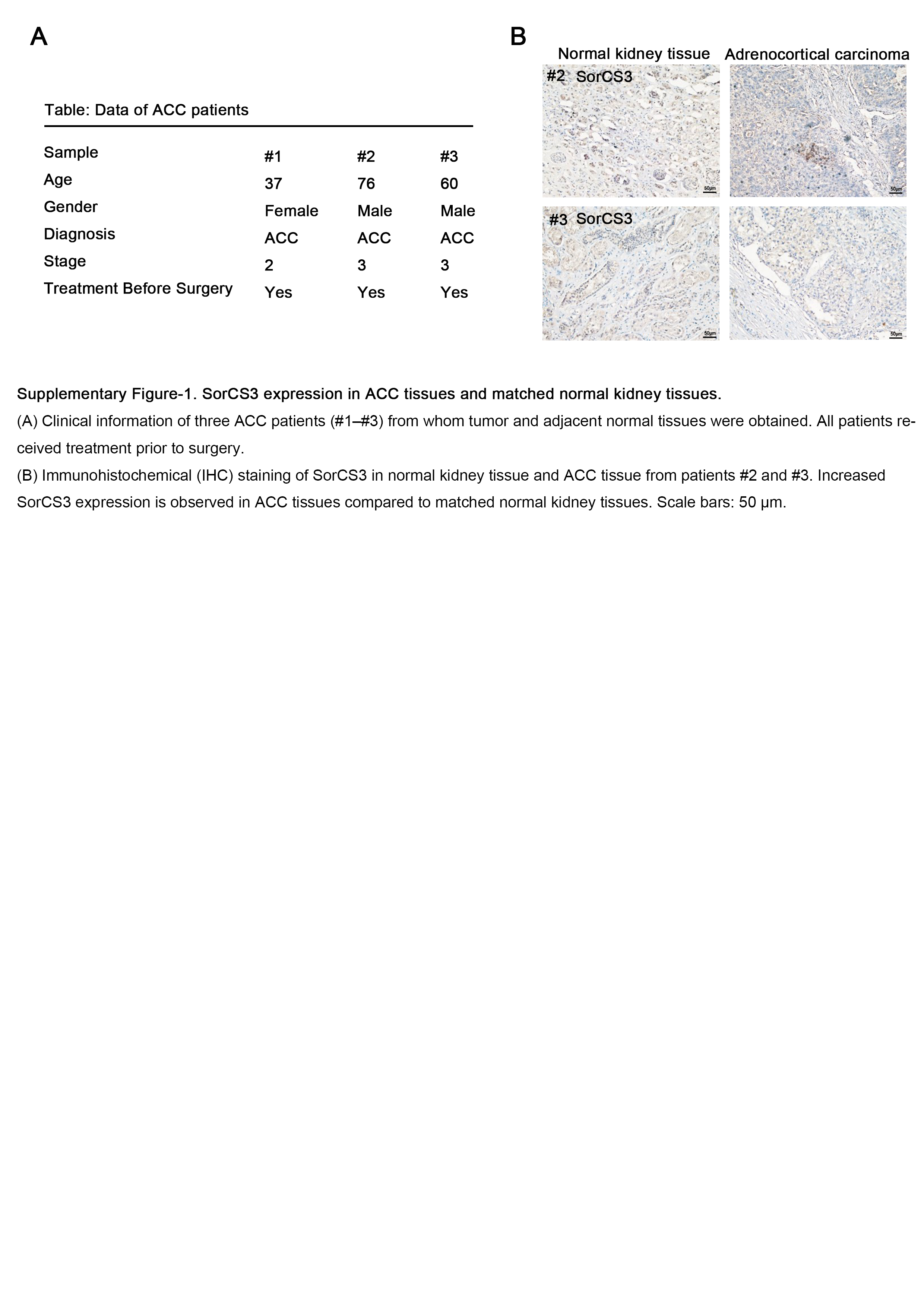

Supplement: Supplementary file 1 — Supplementary Material 1. [file 12967_2025_7146_MOESM1_ESM.tif]

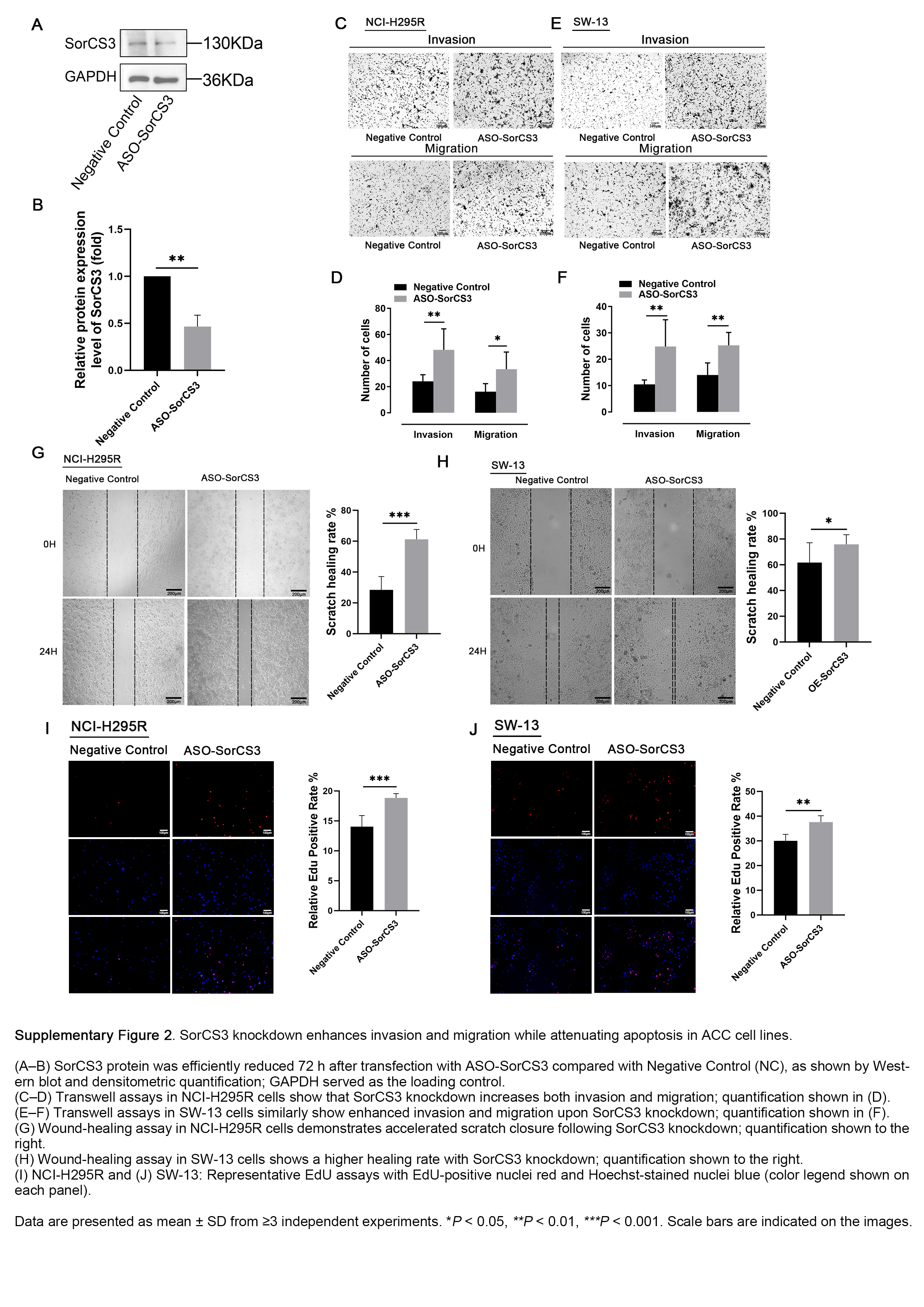

Supplement: Supplementary file 2 — Supplementary Material 2. [file 12967_2025_7146_MOESM2_ESM.tif]

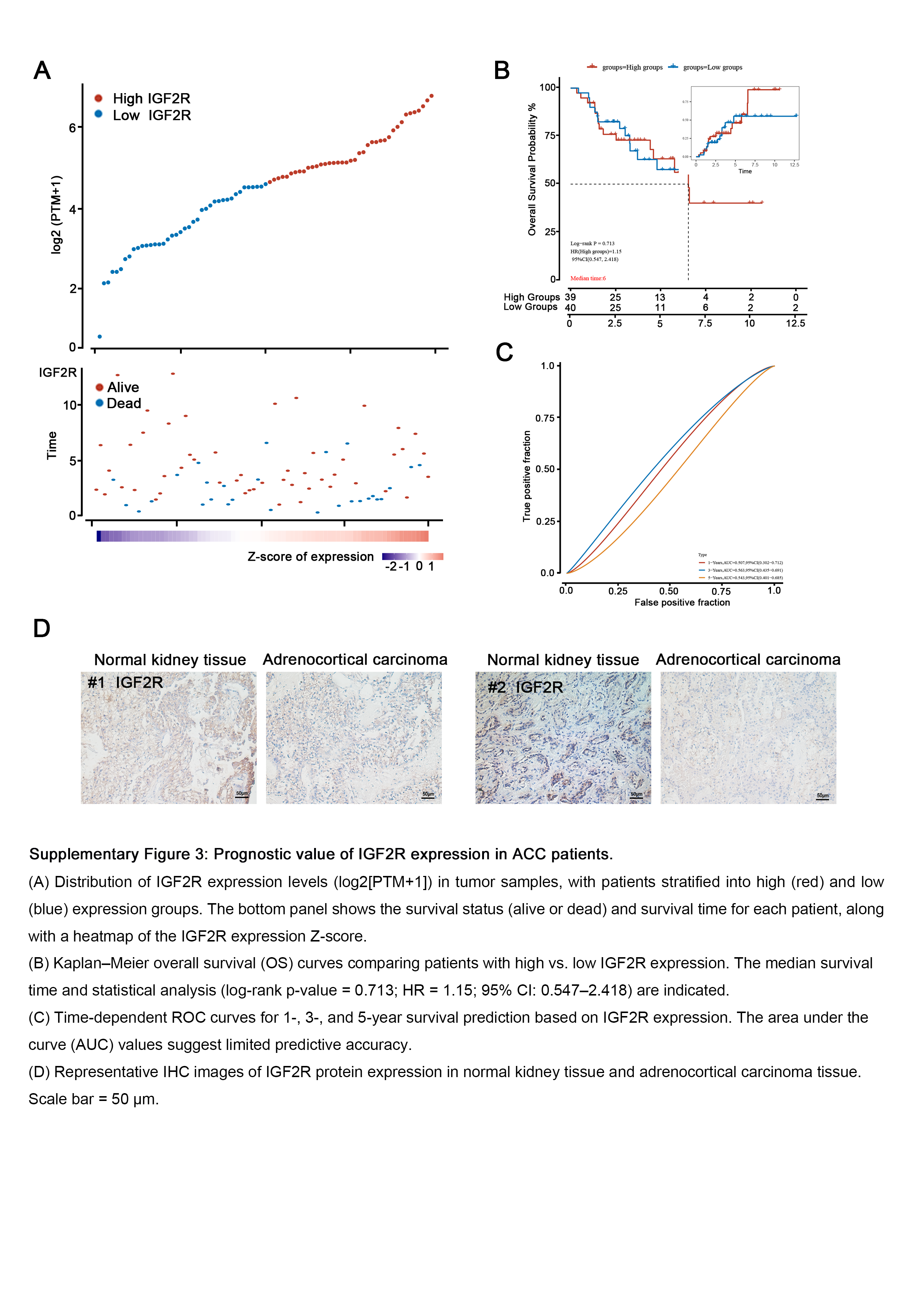

Supplement: Supplementary file 3 — Supplementary Material 3. [file 12967_2025_7146_MOESM3_ESM.tif]

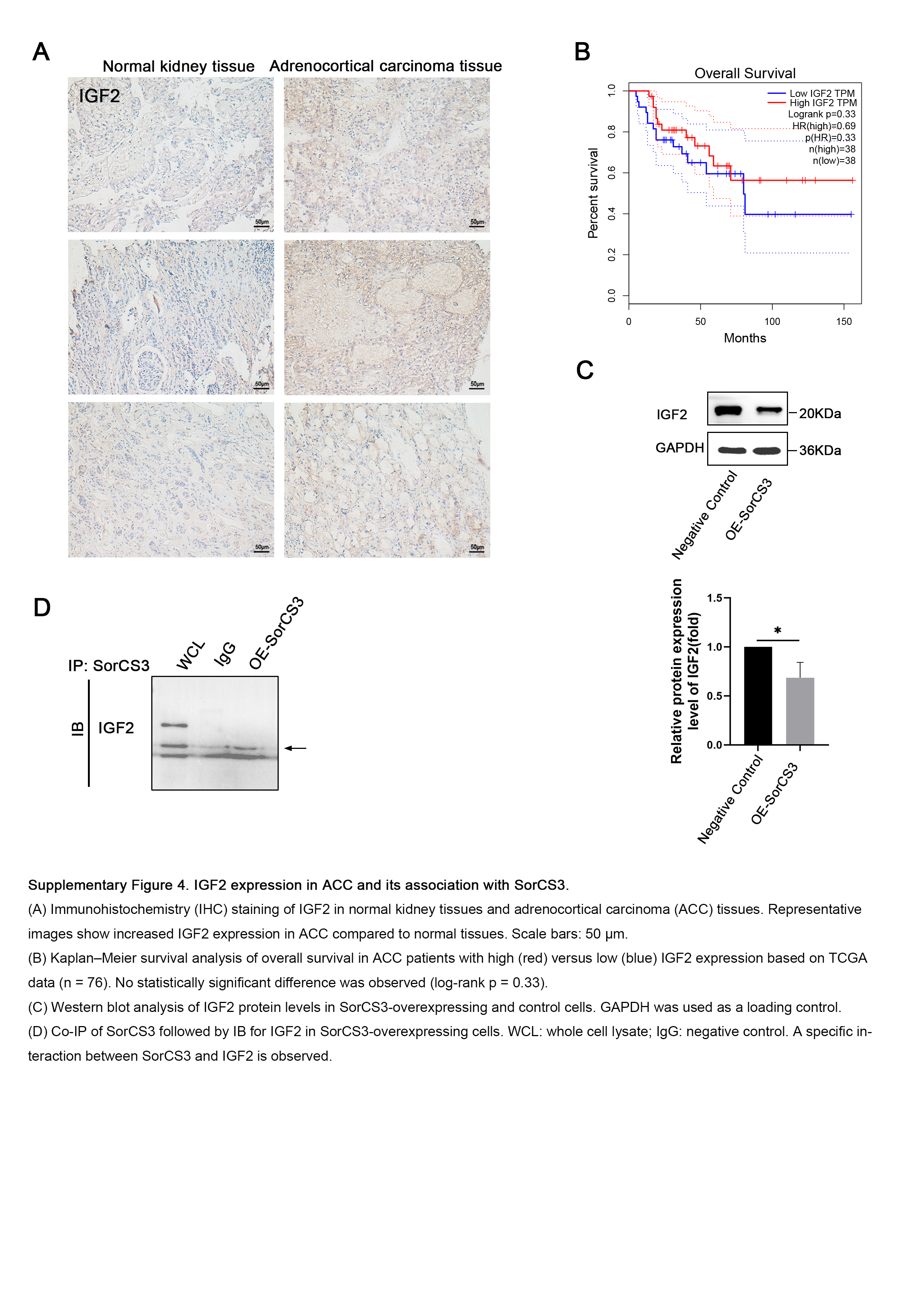

Supplement: Supplementary file 4 — Supplementary Material 4. [file 12967_2025_7146_MOESM4_ESM.tif]

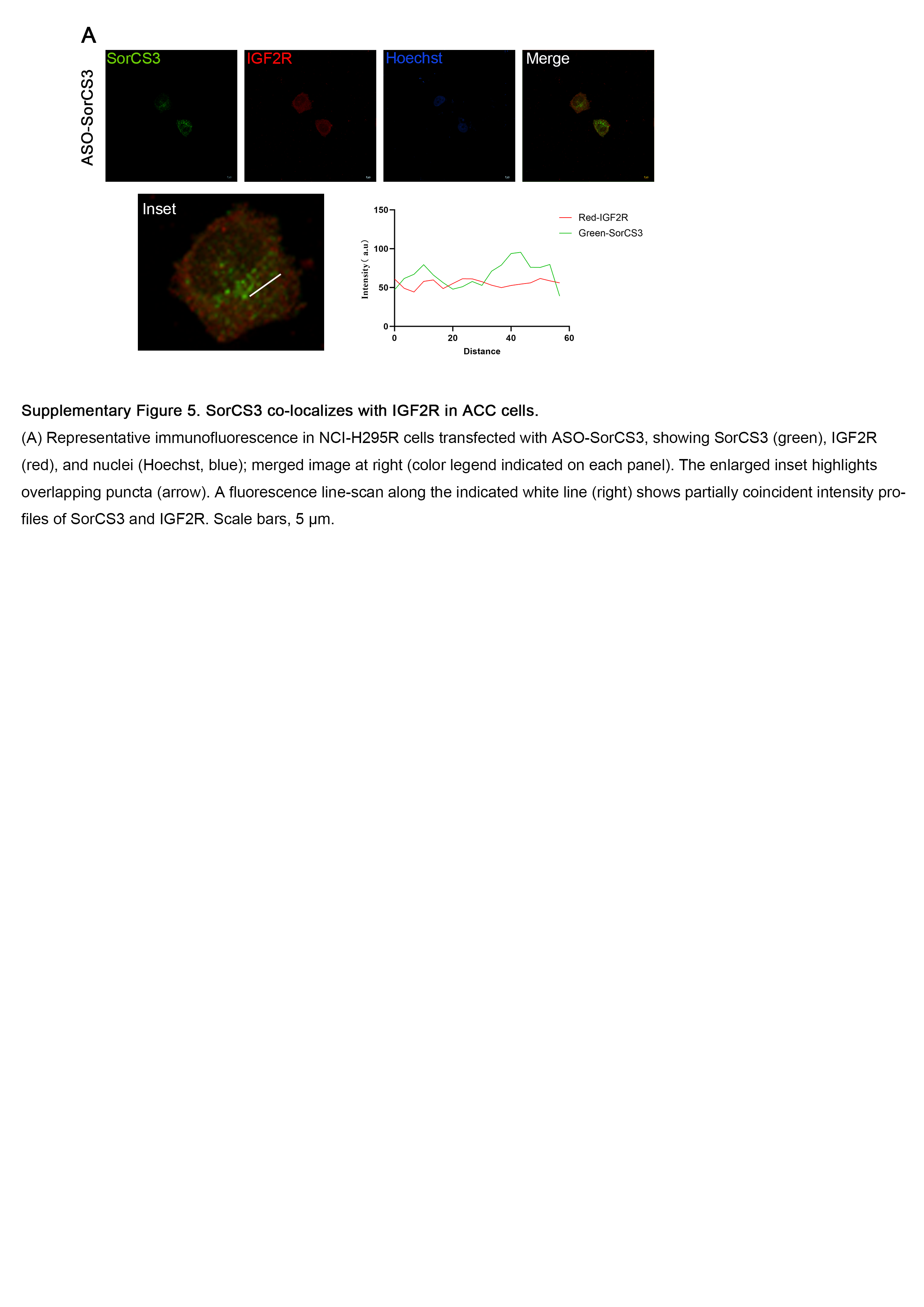

Supplement: Supplementary file 5 — Supplementary Material 5. [file 12967_2025_7146_MOESM5_ESM.tif]

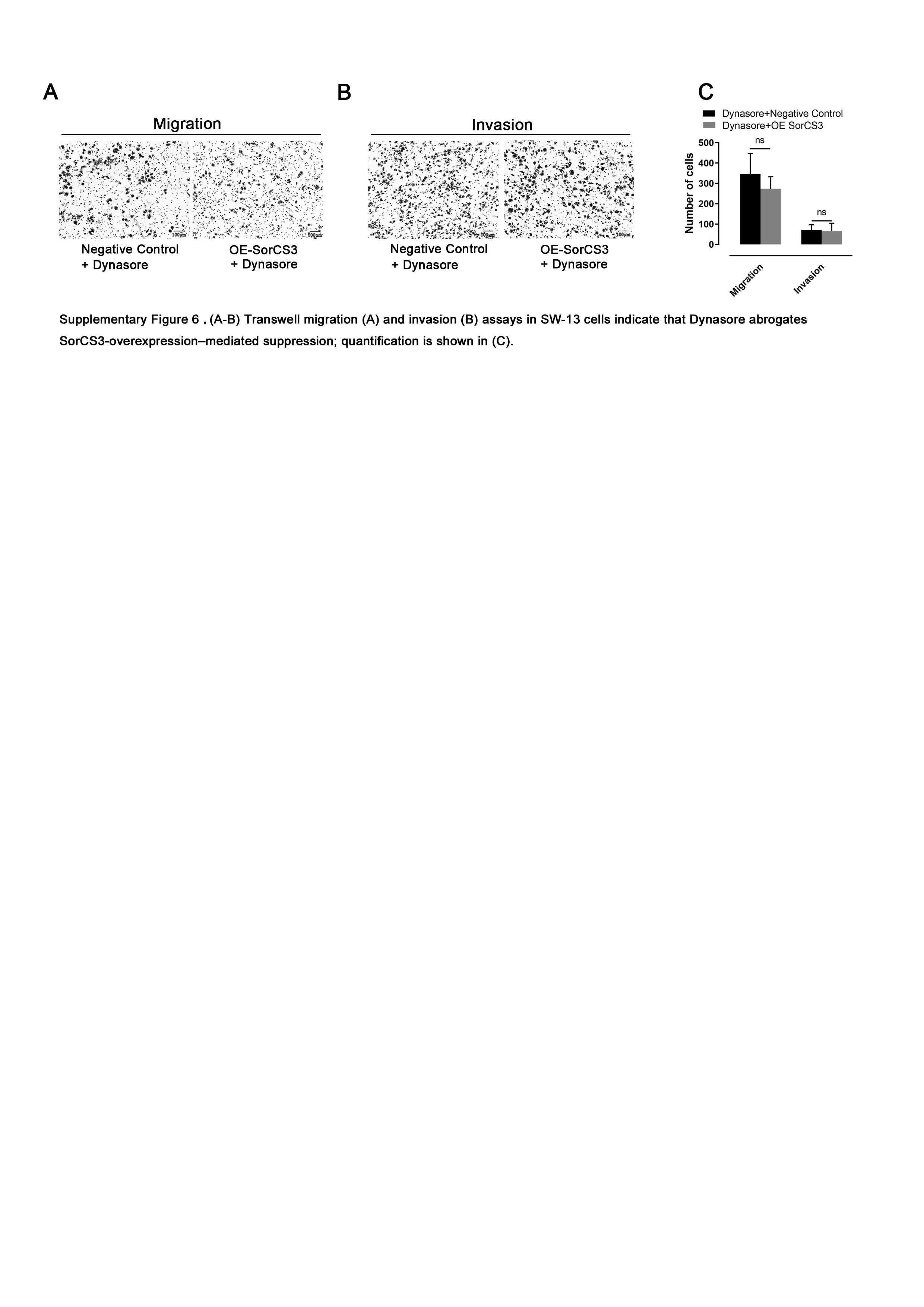

Supplement: Supplementary file 6 — Supplementary Material 6. [file 12967_2025_7146_MOESM6_ESM.tif]

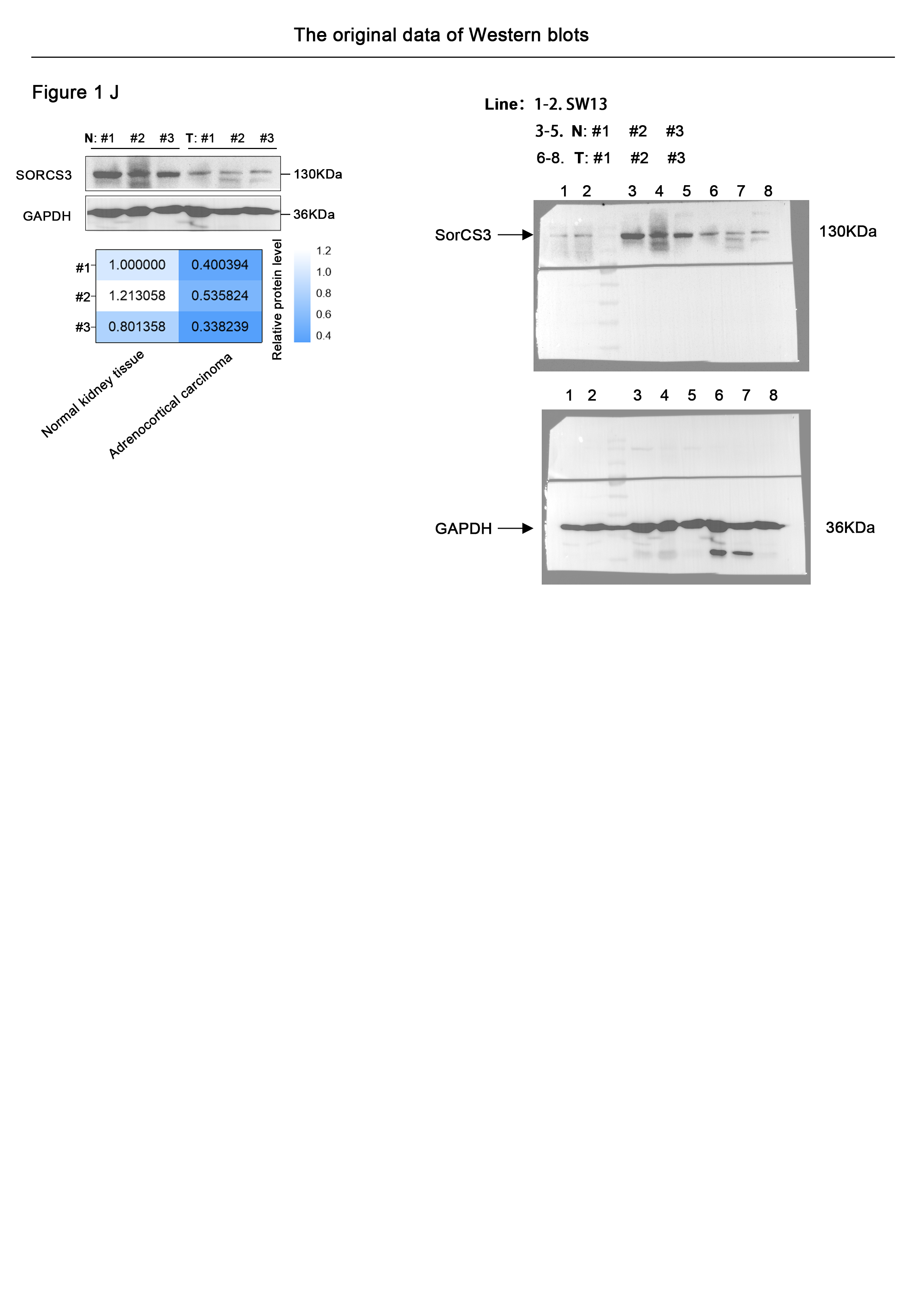

Supplement: Supplementary file 8 — Supplementary Material 8. [file 12967_2025_7146_MOESM8_ESM.tif]

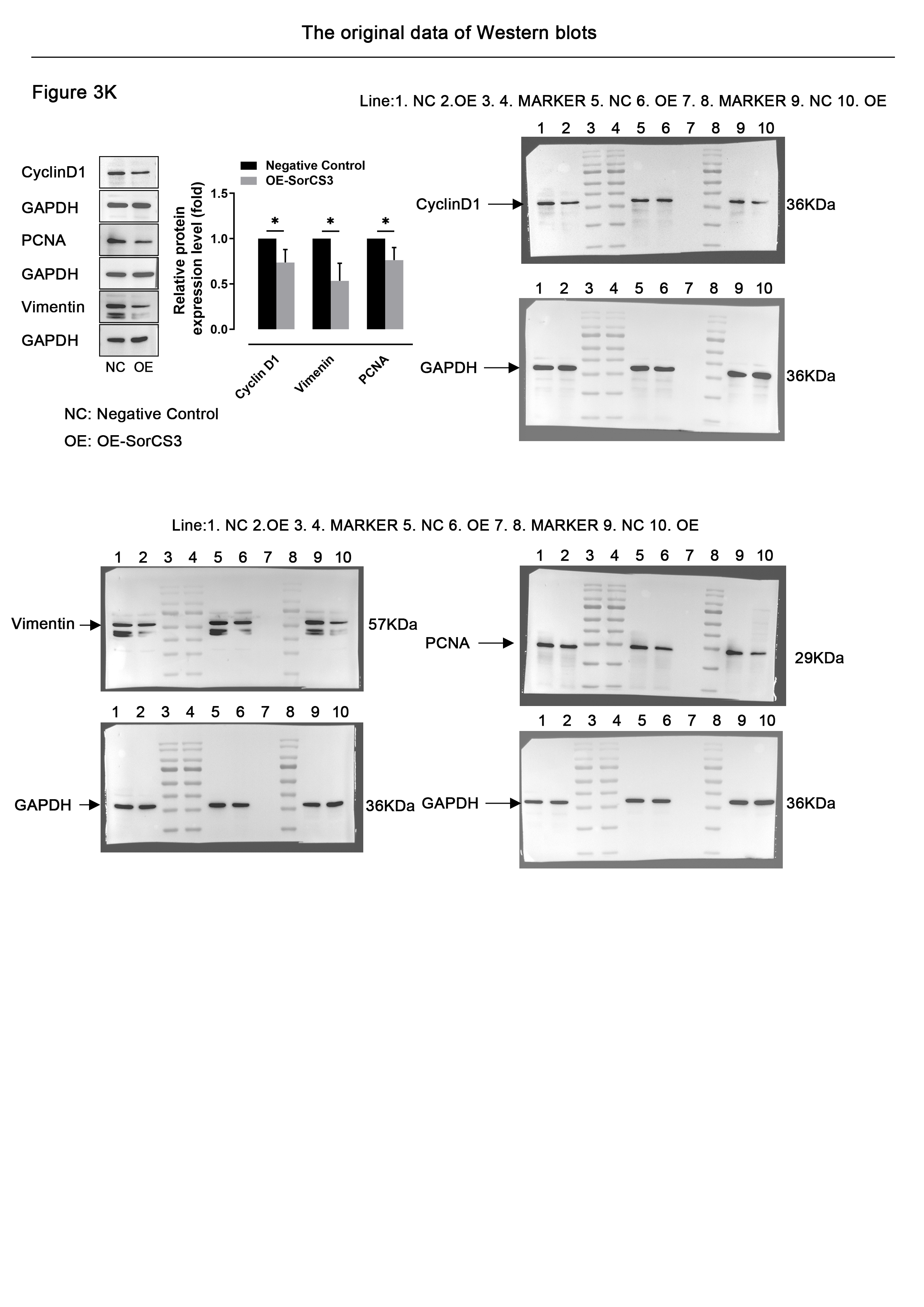

Supplement: Supplementary file 9 — Supplementary Material 9. [file 12967_2025_7146_MOESM9_ESM.tif]

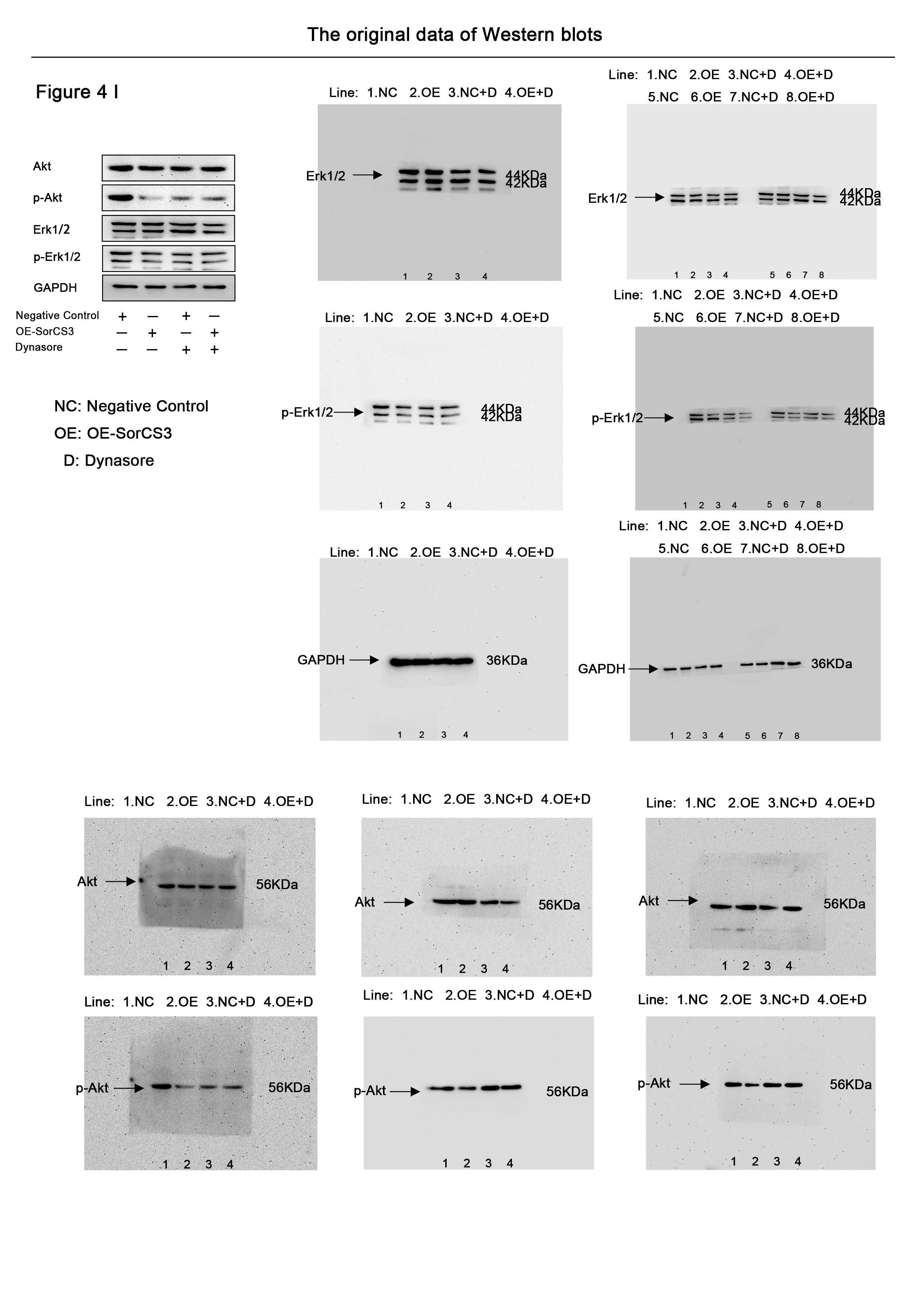

Supplement: Supplementary file 10 — Supplementary Material 10. [file 12967_2025_7146_MOESM10_ESM.tif]

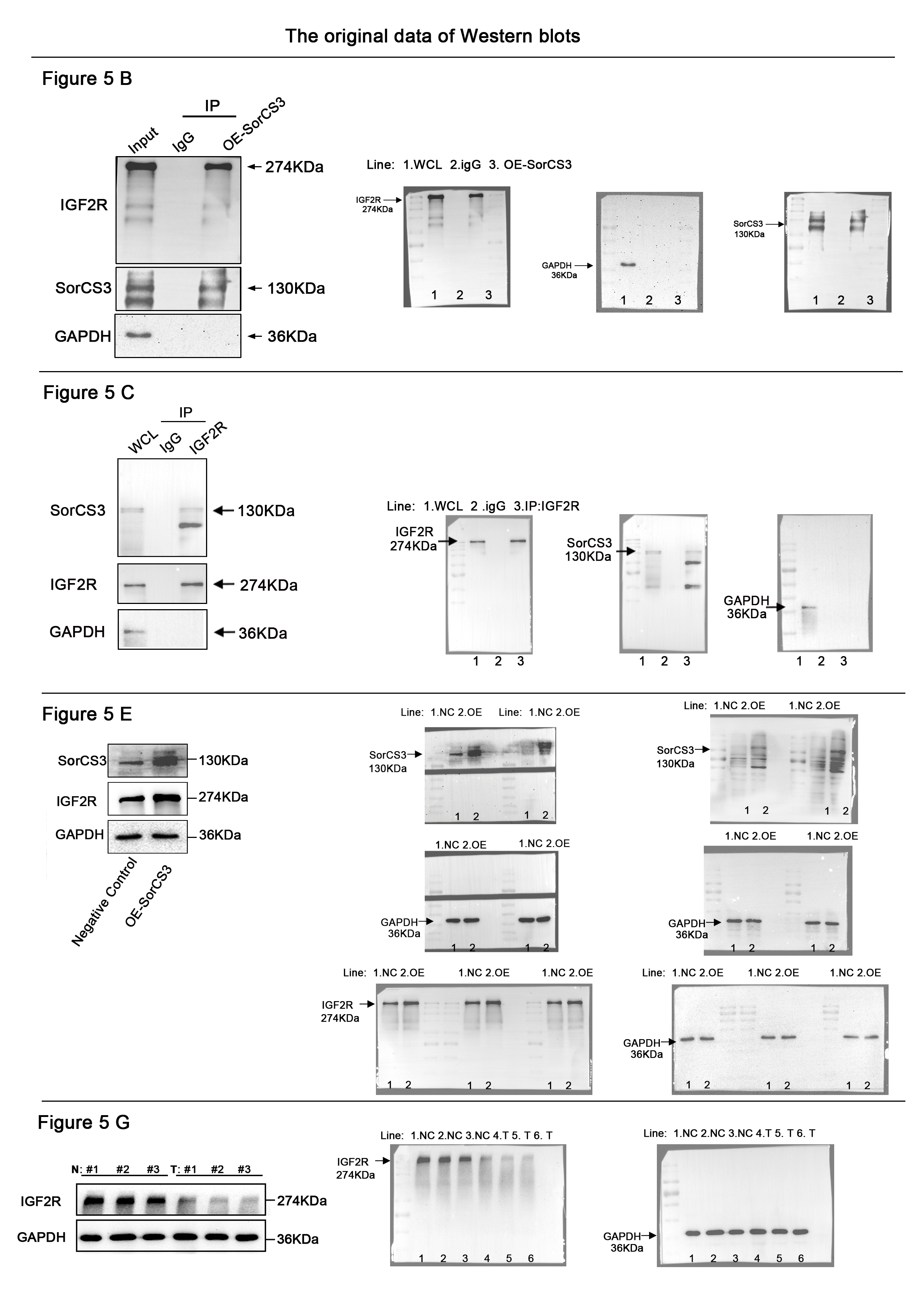

Supplement: Supplementary file 11 — Supplementary Material 11. [file 12967_2025_7146_MOESM11_ESM.tif]
